# CD8^+^ T Cells Cause Disability and Axon Loss in a Mouse Model of Multiple Sclerosis

**DOI:** 10.1371/journal.pone.0012478

**Published:** 2010-08-30

**Authors:** Chandra Deb, Reghann G. LaFrance-Corey, William F. Schmalstieg, Brian M. Sauer, Huan Wang, Christopher L. German, Anthony J. Windebank, Moses Rodriguez, Charles L. Howe

**Affiliations:** 1 Department of Neurology, Mayo Clinic College of Medicine, Rochester, Minnesota, United States of America; 2 Neurosurgery, Mayo Clinic College of Medicine, Rochester, Minnesota, United States of America; 3 Neuroscience, Mayo Clinic College of Medicine, Rochester, Minnesota, United States of America; 4 Neurobiology of Disease PhD Program, Mayo Clinic College of Medicine, Rochester, Minnesota, United States of America; Julius-Maximilians-Universität Würzburg, Germany

## Abstract

**Background:**

The objective of this study was to test the hypothesis that CD8^+^ T cells directly mediate motor disability and axon injury in the demyelinated central nervous system. We have previously observed that genetic deletion of the CD8^+^ T cell effector molecule perforin leads to preservation of motor function and preservation of spinal axons in chronically demyelinated mice.

**Methodology/Principal Findings:**

To determine if CD8^+^ T cells are necessary and sufficient to directly injure demyelinated axons, we adoptively transferred purified perforin-competent CD8^+^ spinal cord-infiltrating T cells into profoundly demyelinated but functionally preserved perforin-deficient host mice. Transfer of CD8^+^ spinal cord-infiltrating T cells rapidly and irreversibly impaired motor function, disrupted spinal cord motor conduction, and reduced the number of medium- and large-caliber spinal axons. Likewise, immunodepletion of CD8^+^ T cells from chronically demyelinated wildtype mice preserved motor function and limited axon loss without altering other disease parameters.

**Conclusions/Significance:**

In multiple sclerosis patients, CD8^+^ T cells outnumber CD4^+^ T cells in active lesions and the number of CD8^+^ T cells correlates with the extent of ongoing axon injury and functional disability. Our findings suggest that CD8^+^ T cells may directly injure demyelinated axons and are therefore a viable therapeutic target to protect axons and motor function in patients with multiple sclerosis.

## Introduction

The identification of cellular and molecular mediators of axon injury during chronic demyelination is critical to understanding and preventing or repairing the loss of function associated with multiple sclerosis (MS) and other demyelinating diseases of the central nervous system. At present, the MS field has defined the following possible mechanisms for axon injury [1]: 1) demyelination is the necessary and sufficient inciting pathology in MS, with axons lost as a direct consequence of failed glial support; 2) demyelination is necessary but not sufficient for axon injury – the loss of myelin exposes axons to a cytotoxic mileu; 3) demyelination is necessary but not sufficient for axon injury – the loss of myelin exposes axons to axopathic immune effectors such as T cells; 4) neurons and axons are the primary locus for the initiation of MS pathology, and demyelination follows the injury of axons. These mechanisms are not mutually exclusive and each is likely to play an overlapping role in some patients, in some lesions, and at some stage of disease evolution. Here we focus on the hypothesis that the loss of myelin is a necessary predisposing factor for axon injury in MS, but demyelination itself is insufficient to cause axon loss. Rather, loss of myelin renders the axolemma accessible to CNS infiltrating immune effectors such as CD8^+^ T cells [2]–[5].

CD8^+^ T cells are the most frequent T cell subset observed in MS lesions, outnumbering CD4^+^ T cells by up to 10-fold at sites of active inflammation [6], [7]. The number of CD8^+^ T cells found within acute lesions correlates very well with the extent of ongoing axon injury exhibited by MS patients [8]. Unlike CD4^+^ T cells in MS lesions, CD8^+^ T cells exhibit clonal expansion, suggesting the recruitment and local proliferation of a distinct, epitope-specific repertoire in the brain [6], [9]–[11]. Importantly, MHC class I is upregulated on axons within active MS lesions [12]. Finally, while anti-CD4 monoclonal antibody treatment failed to alter disease course in MS patients [13], treatment with alemtuzumab [14], a monoclonal antibody that depletes both CD8^+^ and CD4^+^ T cells, or treatment with natalizumab [15], [16], which prevents both CD8^+^ and CD4^+^ T cell trafficking into the CNS, resulted in a reduction in relapse rate.

The cytotoxic mechanisms used by CD8^+^ T cells include cytokine-mediated toxicity, FasL induction of target cell apoptosis, and target cell killing mediated by perforin and granzyme [17]. Recent evidence suggests that elevation of granzyme levels in the CSF may correlate with relapse in MS patients [18], and a considerable literature indicates that CD8^+^ T cells mediate at least some aspects of axon injury in some animal models of MS via a perforin-dependent pathway [5]. Based on our previous observations that demyelinated axons and motor function are protected by the genetic deletion of perforin in the Theiler's murine encephalomyelitis virus (TMEV) model of MS [19]–[22], we have attempted to further define the role of CD8^+^ T cells in the injury of demyelinated axons. Using the adoptive transfer of a population of spinal cord infiltrating, acute effector CD8^+^ T cells that we have recently characterized [23], we now report that CD8^+^ T cells are necessary and sufficient to injure demyelinated axons in vivo in a perforin- and MHC class I-dependent manner.

## Results

### Axons and motor function are preserved in demyelinated perforin-deficient mice

**Figure 1:**
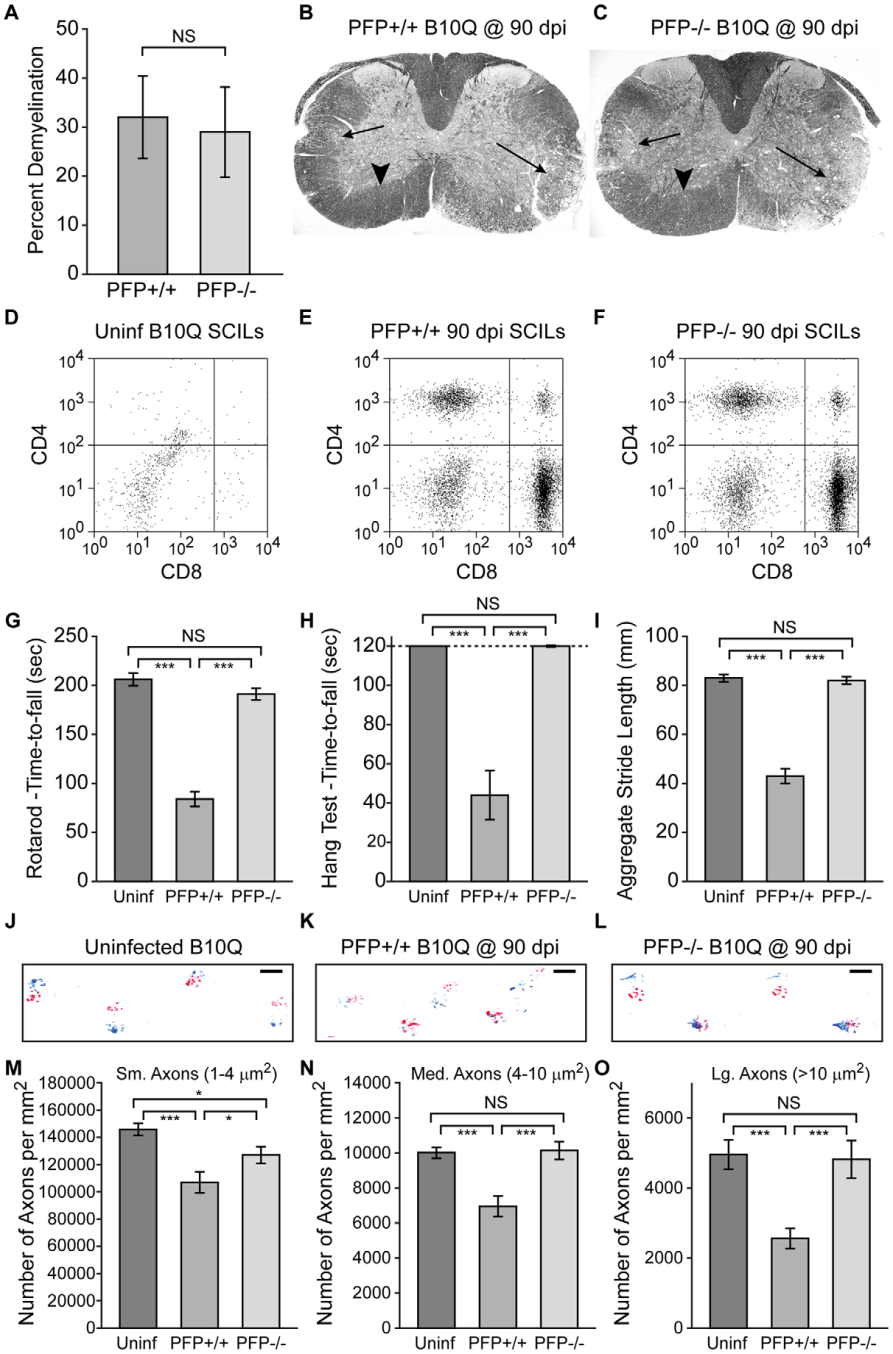
Function and axons are protected in the absence of perforin. Absence of perforin confers protection of motor function and preservation of spinal axons in the demyelinated spinal cord at 90 dpi. All data are presented as mean ±95% C.I. Statistical significance defined as *P<0.05, **P<0.01, ***P<0.001. Significance determined by one-way ANOVA with SNK pairwise posthoc analysis, except demyelination, which was analyzed by t-test. All measurements made at 90 dpi. Uninf  =  uninfected control mice; PFP+/+  =  perforin-competent mice; PFP−/−  =  perforin-deficient mice. (A–C) The percent (A) and distribution (B–C) of spinal cord white matter that was demyelinated did not differ at 90 dpi between perforin-competent (B) or perforin-deficient (C) mice. Arrows indicate demyelinated lesions. Arrowheads indicate normal-appearing white matter. (D–F) Very few CD45^hi^CD4^+^ or CD45^hi^CD8^+^ leukocytes were present in the spinal cord of uninfected mice (D). No difference in the number of CD45^hi^CD4^+^ or CD45^hi^CD8^+^ cells in the spinal cord infiltrating leukocytes (SCILs) was observed between perforin-competent (E) and perforin-deficient (F) mice at 90 dpi. (G–I) Motor function as assessed by rotarod (G), hanging wire test (H), and footprint analysis (I) was significantly impaired in perforin-competent mice at 90 dpi but was preserved in perforin-deficient mice at the same time point. (J–L) Representative examples of stride. Scale bar represent 10 mm. (M–O) As with function, small- (M), medium- (N), and large- (O) caliber axons were preserved in perforin-deficient mice as compared to perforin-competent mice at 90 dpi. Medium- and large-caliber axons were preserved to uninfected control levels in perforin-deficient mice.

Perforin-competent and perforin-deficient mice on an MHC H-2^q^ background (B10Q) were infected with 2×10^5^ PFU TMEV by intracranial injection at 4–6 weeks of age to induce demyelinating disease. At 90 days postinfection (dpi), we found that demyelinated lesion load (Figure 1A) and lesion distribution (Figure 1B–C) were the same irrespective of perforin expression (P = 0.645 by t-test; Table S1 contains detailed statistical analysis for all measurements). Likewise, viral load in the spinal cord at 90 dpi did not differ between perforin-competent (5.45±0.37 log_10_ viral copies; all data are presented as mean±95% CI) and perforin-deficient mice (5.26±0.49 log_10_ copies; P = 0.505 by Mann-Whitney rank sum test). We also found that the number of CD4^+^ and CD8^+^ T cells present in a purified population of spinal cord-infiltrating leukocytes (SCILs) [23] collected at 90 dpi did not differ based on perforin expression (Figure 1D–F). SCILs from perforin-competent mice at 90 dpi contained 1492±122 CD45^hi^CD8^+^ cells and 339±33 CD45^hi^CD4^+^ cells, while SCILs from perforin-deficient mice at 90 dpi contained 1495±183 CD45^hi^CD8^+^ cells (P = 0.976 by t-test) and 369±80 CD45^hi^CD4^+^ cells (P = 0.523 by t-test) (Figure S1).

However, despite identical disease progression [19], motor function in perforin-deficient mice at 90 dpi was remarkably preserved relative to perforin-competent mice at the same time point. In fact, demyelinated perforin-deficient mice were indistinguishable from uninfected, normally myelinated age-matched controls by rotarod (P = 0.104) (Figure 1G), hanging wire test (P = 0.942) (Figure 1H), and footprint analysis (P = 0.948) (Figure 1I), whereas demyelinated perforin-competent mice under identical conditions were severely and significantly impaired (P<0.001 vs perforin-competent and vs uninfected for all measurements) (Figure 1G–I). Preservation of function in demyelinated perforin-deficient mice, as compared to demyelinated perforin-competent mice, was apparent by stride length and footstep positioning (Figure 1J-L), by open-field video analysis (Movie S1), by runway video analysis (Movie S2), and by direct observation of posture (Figure S2).

Loss of function in perforin-competent mice and preservation in perforin-deficient mice was mirrored in the number of intact axons present in the thoracic spinal cord at 90 dpi [19]–[21]. We counted the absolute number of small- (1–4 µm^2^), medium (4–10 µm^2^), and large- (>10 µm^2^) caliber axons in plastic-embedded ultrathin sections of thoracic spinal cord stained with 4% para-phenylenediamine [19]. There was no significant difference in the number of medium- or large-caliber spinal axons between uninfected control mice and perforin-deficient mice at 90 dpi (P = 0.994 for medium axons; P = 0.762 for large axons) (Figure 1M–O). In contrast, in perforin-competent mice at 90 dpi the number of medium-caliber axons was reduced to 68% of perforin-deficient levels (P<0.001) and the number of large-caliber axons was reduced to 53% of perforin-deficient levels (P<0.001) (Figure 1M–O). At the same time, small-caliber axons were reduced to 73% of uninfected levels in perforin-competent mice (P<0.001) and to 87% of uninfected levels in perforin-deficient mice (P = 0.033). However, despite the reduction of small axons in perforin-deficient mice, there was still relative preservation as compared to perforin-competent mice (P = 0.031). Thus, we conclude that the absence of perforin in B10.Q mice protects motor function and spinal axons at 90 dpi despite demyelinated lesion loads, viral loads, and a SCILs population that are indistinguishable from perforin-competent mice at the same time point.

### MHC class I is expressed on axons in demyelinated lesions

**Figure 2:**
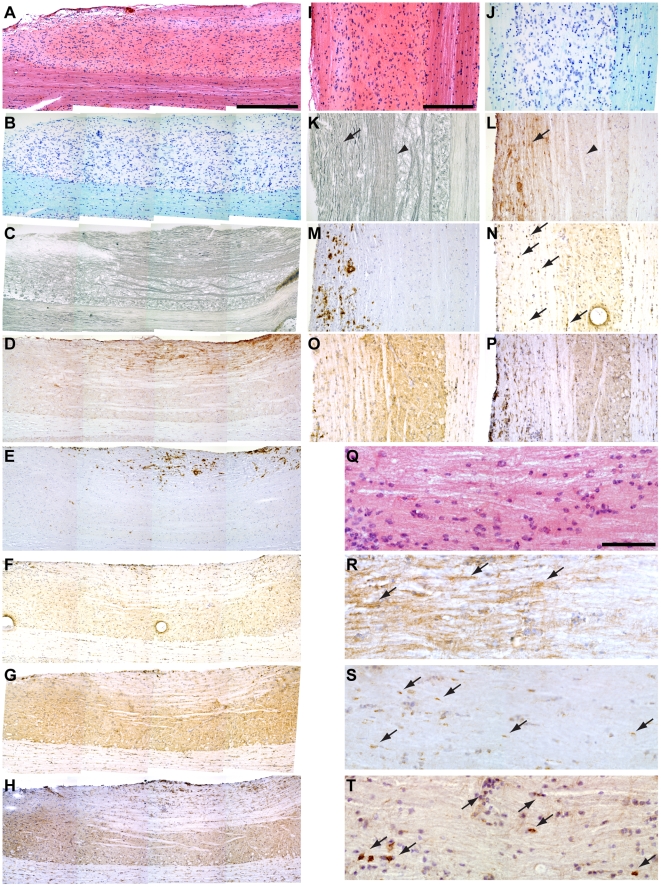
MHC class I is expressed in demyelinated lesions. MHC class I expression is localized to sites of demyelination, axon injury, and CD8^+^ T cell infiltration. (A–C, I–K, Q) Histology of spinal cord at 90 dpi revealed cellular infiltrate by H&E (A, I, Q), loss of Luxol fast blue stained myelin (B, J), and dysmorphic Bielschowsky-stained axons (C, K). (D–H, L–P, R–T) Immunostaining further revealed MHC class I expression on fibers in the demyelinated lesion (D, L, R), MHC class II staining on cells in the demyelinated lesion (E, M), and the presence of CD8^+^ (F, N, T), CD4^+^ (G, O), and CD11b^+^ (H, P) immune cells within the demyelinated lesion. (S) The axon injury marker APP was also observed on structures within demyelinated lesions containing MHC class and CD8^+^ cells. Scale bar in (A) is 500 µm and refers to (A–H). Scale bar in (I) is 250 µm and refers to (I–P). Scale bar in (Q) is 50 µm and refers to (Q–T).

**Figure 3:**
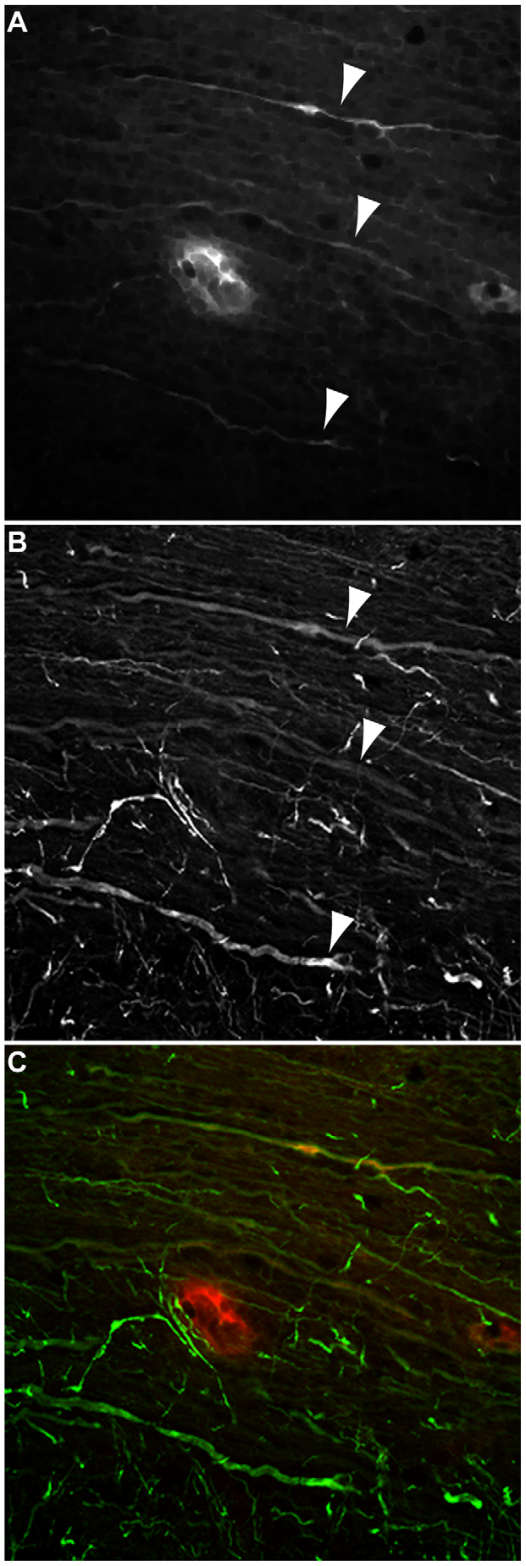
MHC class I is expressed on axons in demyelinated lesions. MHC class I expression colocalizes with dysmorphic axons immunostained for neurofilament. (A, C) MHC class I expression was observed in the 90 dpi spinal cord on fiber-like structures (red channel in C). (B, C) These same structures expressed neurofilaments and exhibited a dysmorphic pattern consistent with injured axons (green channel in C). Arrowheads point to the same structures in (A) and (B).

Our observations in perforin-deficient B10.Q mice suggested a primary role for CD8^+^ T cells in axon injury and loss of motor function. A critical component of any model involving CD8^+^ T cells is expression of cognate MHC class I molecules on the target structure. Histological and immunohistochemical analyses revealed MHC class I expression on longitudinally transverse structures within demyelinated lesions of the spinal cord at 90 dpi (Figure 2). Demyelinated lesions identified by H&E (Figure 2A, 2I), Luxol fast blue (Figure 2B, 2J), and Bielschowsky (Figure 2C, 2K) staining were found to contain MHC class I- (Figure 2D, 2L) and MHC class II-positive (Figure 2E, 2M) structures. The MHC class I staining was suggestive of fibers while the MHC class II staining exhibited a predominantly cellular profile. Within the same lesions and in proximity to MHC class I- and II-positive structures, we observed CD8- (Figure 2F, 2N), CD4- (Figure 2G, 2O), and CD11b-positive (Figure 2H, 2P) cells. Higher magnification analysis of a demyelinated lesion (Figure 2Q) revealed many MHC class I-positive fiber-like structures (Figure 2R) spatially associated with accumulations of the axon injury marker APP (Figure 2S) and in proximity to CD8-positive immune cells (Figure 2T). Finally, double-labeling immunofluorescence analysis of demyelinated lesions in the spinal cord provided evidence of MHC class I-positive (Figure 3A, 3C) neurofilament-containing fibers (Figure 3B) exhibiting structural characteristics consistent with injured axons. We conclude that demyelinated axons in the B10.Q spinal cord at 90 dpi have the requisite MHC class I expression necessary to interact with CD8^+^ T cells.

### Adoptive transfer of perforin-competent SCILs ablates host axons and function

**Figure 4:**
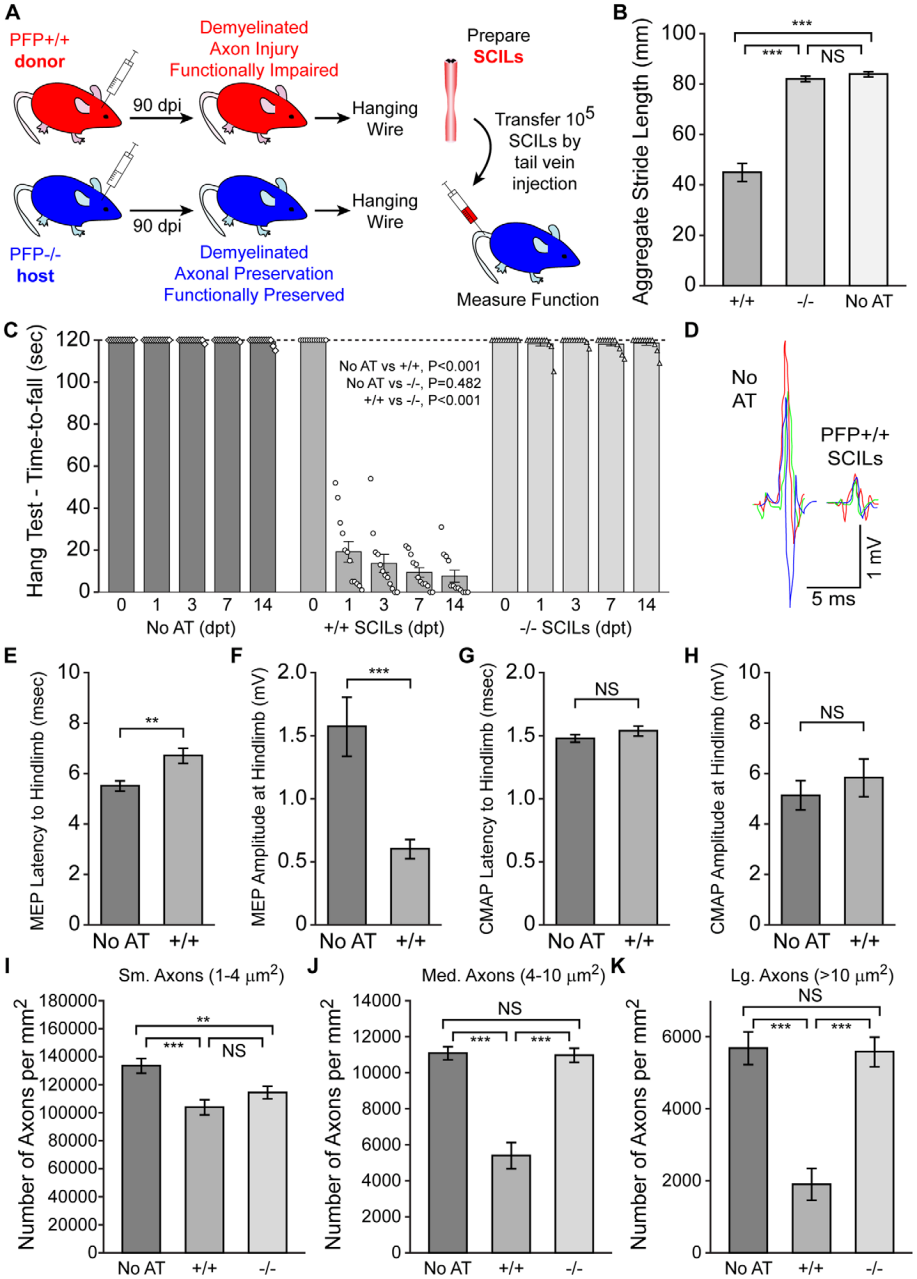
Function and axons are lost following adoptive transfer of perforin-competent cells. Adoptive transfer of perforin-competent spinal cord infiltrating leukocytes (SCILs) into demyelinated but functionally preserved perforin-deficient hosts induces rapid and permanent (>1 mo) loss of motor function and spinal axons. All data are presented as mean ±95% C.I. Statistical significance defined as *P<0.05, **P<0.01, ***P<0.001. Significance determined by one-way ANOVA with SNK pairwise posthoc analysis, except electrophysiology, which was analyzed by t-test, and the hanging wire test, which was analyzed by two-way ANOVA. All measurements made at 7 days post-transfer (dpt) unless otherwise indicated. No AT  =  no adoptive transfer controls; +/+  =  adoptive transfer of perforin-competent SCILs; −/−  =  adoptive transfer of perforin-deficient SCILs. (A) Schematic representation of the adoptive transfer paradigm. (B) Adoptive transfer of perforin-competent SCILs into perforin-deficient hosts at 90 dpi results in significant loss of function as assessed by footprint analysis. In contrast, adoptive transfer of perforin-deficient SCILs into perforin-deficient hosts causes no loss of function. (C) Longitudinal analysis of motor function by the hanging wire test shows that adoptive transfer of perforin-competent SCILs into 90 dpi perforin-deficient hosts results in a very rapid loss of function that does not recover over the analyzed time frame. Each symbol represents an individual animal. (D) Representative motor evoked potentials (MEP) in perforin-deficient 90 dpi hosts receiving either no adoptive transfer or perforin-competent SCILs adoptive transfer. (E-H) Central motor conduction latency (E) and amplitude (F) were significantly altered by adoptive transfer of perforin-competent SCILs as compared to perforin-deficient mice at 90 dpi that received no adoptive transfer. In contrast, peripheral compound motor action potential (CMAP) latency (G) and amplitude (H) were unaffected by adoptive transfer. (I-K) Adoptive transfer of perforin-competent SCILs into 90 dpi perforin-deficient hosts resulted in significant loss of small- (I), medium- (J), and large- (K) caliber spinal axons as compared to perforin-deficient 90 dpi hosts that received no adoptive transfer. Transfer of perforin-deficient SCILs into perforin-deficient hosts caused no loss of medium- (J) or large- (K) caliber axons, but did induce a signficant reduction in small- (I) caliber axons.

To directly test our hypothesis regarding the role of CD8^+^ T cells in the injury of demyelinated axons, we established an adoptive transfer strategy that allowed us to introduce perforin-competent SCILs into demyelinated but functionally preserved perforin-deficient mice (Figure 4A). Our goal was to transiently reconstitute perforin-deficient hosts with perforin-competent leukocytes that had homed to the spinal cord of demyelinated donor mice experiencing active axon injury. For these experiments, perforin-deficient hosts at 90 dpi received either a sham adoptive transfer (RPMI used to resuspend SCILs  =  “No AT”), perforin-competent SCILs collected from the spinal cord of donor perforin wildtype B10Q mice at 90 dpi (donor cohorts were infected at the same time as host cohorts and were assessed for motor function in parallel with the perforin-deficient hosts), or perforin-deficient SCILs collected from perforin knock-out hosts at 90 dpi. Donor SCILs were counted, phenotyped by flow cytometry, and injected into the tail vein of host perforin-deficient mice (10^5^ SCILs per host for most experiments). As previously published [23], for every 10^5^ cells adoptively transferred, approximately 10% were CD45^hi^ and 5% were CD45^hi^CD8^+^.

At 7 days post-transfer (dpt), we found that hosts that received perforin-competent SCILs showed a 50% reduction in aggregate stride length as compared to demyelinated perforin-deficient hosts that received sham adoptive transfer (P<0.001) (Figure 4B). In contrast, perforin-deficient hosts that received perforin-deficient SCILs were unimpaired (P = 0.658 vs sham AT; P<0.001 vs perforin-competent AT) (Figure 4B). Moreover, using the hanging wire test we observed that adoptive transfer of perforin-competent SCILs into demyelinated perforin-deficient hosts ablated motor function by 24 hr post-transfer (Figure 4C). This loss of function persisted through 14 days (Figure 4C) and has never been seen to recover within the 2 months that we have followed mice after adoptive transfer. Loss of function following adoptive transfer of perforin-competent SCILs was also apparent by runway video analysis (Movie S3). Adoptive transfer of perforin-deficient SCILs did not cause loss of function in hosts at any time point. We conclude from these observations that demyelinated perforin-deficient hosts are susceptible to rapid and permanent loss of motor function following adoptive transfer of perforin-competent SCILs but not following transfer of perforin-deficient SCILs. This strongly suggests that other axopathic and cytotoxic effector mechanisms present in the leukocytes collected from 90 dpi perforin-deficient donors are unable to damage demyelinated axons and abrogate motor function in our model.

Adoptive transfer of perforin-competent SCILs into perforin-deficient hosts at 90 dpi did not alter viral load or demyelination. Perforin-deficient hosts at 7 days after sham adoptive transfer had 5.61±0.20 log_10_ viral copies, compared to 5.48±0.34 log_10_ viral copies following adoptive transfer of perforin-competent SCILs (P = 0.552 by t-test). Perforin-deficient hosts at 7 days after adoptive transfer of perforin-deficient SCILs exhibited 29%±4.4% spinal demyelination, compared to 27%±6.1% spinal demyelination following adoptive transfer of perforin-competent SCILs (P = 0.349 by t-test).

The adoptive transfer effect depended upon spinal cord demyelination, as transfer of perforin-competent SCILs into uninfected B10Q hosts did not alter aggregate stride length or hanging wire performance. Uninfected hosts receiving sham adoptive transfer had an aggregate stride of 81±1.9 mm and a time-to-fall of 118±3 sec, compared to a stride length of 83±3.4 mm and a time-to-fall of 120±0 sec in uninfected hosts receiving perforin-competent SCILs (footprint: P = 0.303 by t-test; hanging wire: P = 0.548 by Mann-Whitney rank sum test). Likewise, the adoptive transfer-induced loss of motor function only occurred when SCILs were used as effectors. Adoptive transfer of 10^5^ splenocytes from 90 dpi perforin-competent B10Q donors or 10^5^ lymphokine-activated perforin-competent killer cells (IL-2 stimulated in vitro) failed to affect motor function in demyelinated perforin-deficient hosts (P = 0.675 by one-way ANOVA for splenocyte or LAK transfer vs control). Likewise, in agreement with our previous observations regarding the lack of anti-viral responses in the SCILs at 90 dpi [23], adoptive transfer of up to 10^6^ splenocytes isolated from perforin-competent B10Q donors subcutaneously immunized with killed TMEV failed to alter motor function in demyelinated perforin-deficient hosts (P = 0.869 by t-test for splenocytes vs control).

Using evoked motor potentials to assess spinal axon function, we found that adoptive transfer of perforin-competent SCILs into demyelinated perforin-deficient hosts resulted in striking disruption of spinal conduction from the high cervical cord (stimulation site) to the gastrocnemius muscle (recording site) (Figure 4D). Compared to perforin-deficient hosts at 90 dpi that received no adoptive transfer, hosts that received perforin-competent SCILs showed a 3-fold reduction in motor evoked potential (MEP) amplitude at 7 days post-transfer (no adoptive transfer vs SCILs transfer, P<0.001 by t-test) (Figure 4D, 4F). Longitudinal analysis of MEP failure rate (defined as the number of motor cortical stimulations that failed to elicit gastrocnemius muscle responses per the total number of stimulations) in the same perforin-deficient mice before adoptive transfer (26%±16% failure) as compared to the failure rate after transfer of perforin-competent SCILs (75%±16% failure) revealed that far fewer potentials were transmitted along the spinal cord (P<0.001 by paired t-test). Of those potentials that were stimulated, the transmission latency was increased from 5.4±0.3 msec (no adoptive transfer) to 7.4±0.8 msec (SCILs transfer) (P = 0.004 by t-test) (Figure 4E). The transmission defect was restricted to the spinal cord, as peripheral compound motor action potentials (CMAP) from the sciatic notch to the gastrocnemius muscle did not differ with regard to latency (P = 0.260) (Figure 4G) or amplitude (P = 0.477) (Figure 4H) between controls and mice receiving perforin-competent SCILs.

Consistent with the observed defect in spinal axon conduction, we found that adoptive transfer of perforin-competent SCILs into perforin-deficient demyelinated hosts at 90 dpi led to a 22%±8.1% reduction in small-caliber spinal axons (P<0.001 vs no transfer) (Figure 4I), a 51%±13% reduction in medium-caliber axons (P<0.001 vs no transfer) (Figure 4J), and a 67%±15% reduction in large-caliber axons (P<0.001 vs no transfer) (Figure 4K). In contrast, adoptive transfer of perforin-deficient SCILs into perforin-deficient hosts at 90 dpi had no significant impact on either medium-caliber (P = 0.847 vs no transfer) or large-caliber axons (P = 0.859 vs no transfer). Despite the complete preservation of medium and large axons, there was a 14% reduction in small-caliber spinal axons following transfer of perforin-deficient SCILs (P = 0.008 vs no transfer). These observations suggest that medium- and large-caliber demyelinated axons are directly injured in a perforin-dependent manner by SCILs, whereas small-caliber axons may be injured by a perforin-independent mechanism.

### Adoptively transferred SCILs home to the spinal cord but not to the cervical lymph nodes or the brain

**Figure 5:**
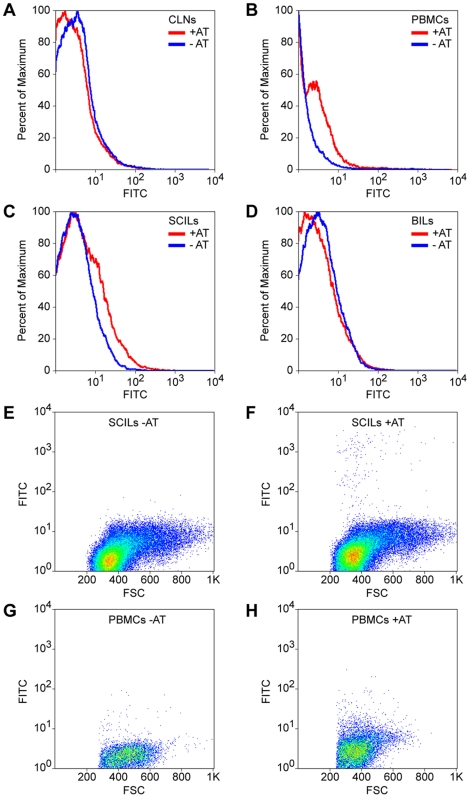
Adoptively transferred SCILs enter the spinal cord. CFSE-labeled SCILs are recovered from the blood (PBMCs) and spinal cord (SCILs) 60 min after adoptive transfer via tail vein injection. (A) Cervical lymph nodes (CLNs) did not contain green CFSE-labeled cells. (B) Green cells were recovered in the blood. (C) Green cells were also recovered from the spinal cord after adoptive transfer. (D) No labeled cells were found in the brain after transfer. (A–D) Red histograms represent the number of cell events observed in hosts that received adoptive transfer of CFSE-labeled SCILs while blue histograms represent control populations collected from mice that did not receive adoptive transfer. (E–H) Representative flow plots showing green cells isolated from the spinal cord (F) and blood (H) of hosts receiving CFSE-labeled SCILs, as compared to control spinal cord (E) and blood (G).

To identify the primary locus of effect for the adoptively transferred SCILs we labeled the cells with CFSE prior to transfer and then recovered SCILs, brain-infiltrating lymphocytes, cervical lymph node cells, and peripheral blood mononuclear cells (PBMCs) at 1 hr post-transfer. Flow cytometry of the isolated cell preparations revealed that relative to sham transfer animals, adoptive transfer hosts had small populations of CFSE-labeled cells in the blood (Figure 5B, 5G, 5H) and in the SCILs (Figure 5C, 5E, 5F) but not in the cervical lymph nodes (Figure 5A) or the brain (Figure 5D). While further experiments are required to define the kinetics of infiltration into the CNS, we conclude that adoptively transferred SCILs are competent to enter the demyelinated spinal cord.

### CD8^+^ T cells are necessary and sufficient to injure demyelinated axons

**Figure 6:**
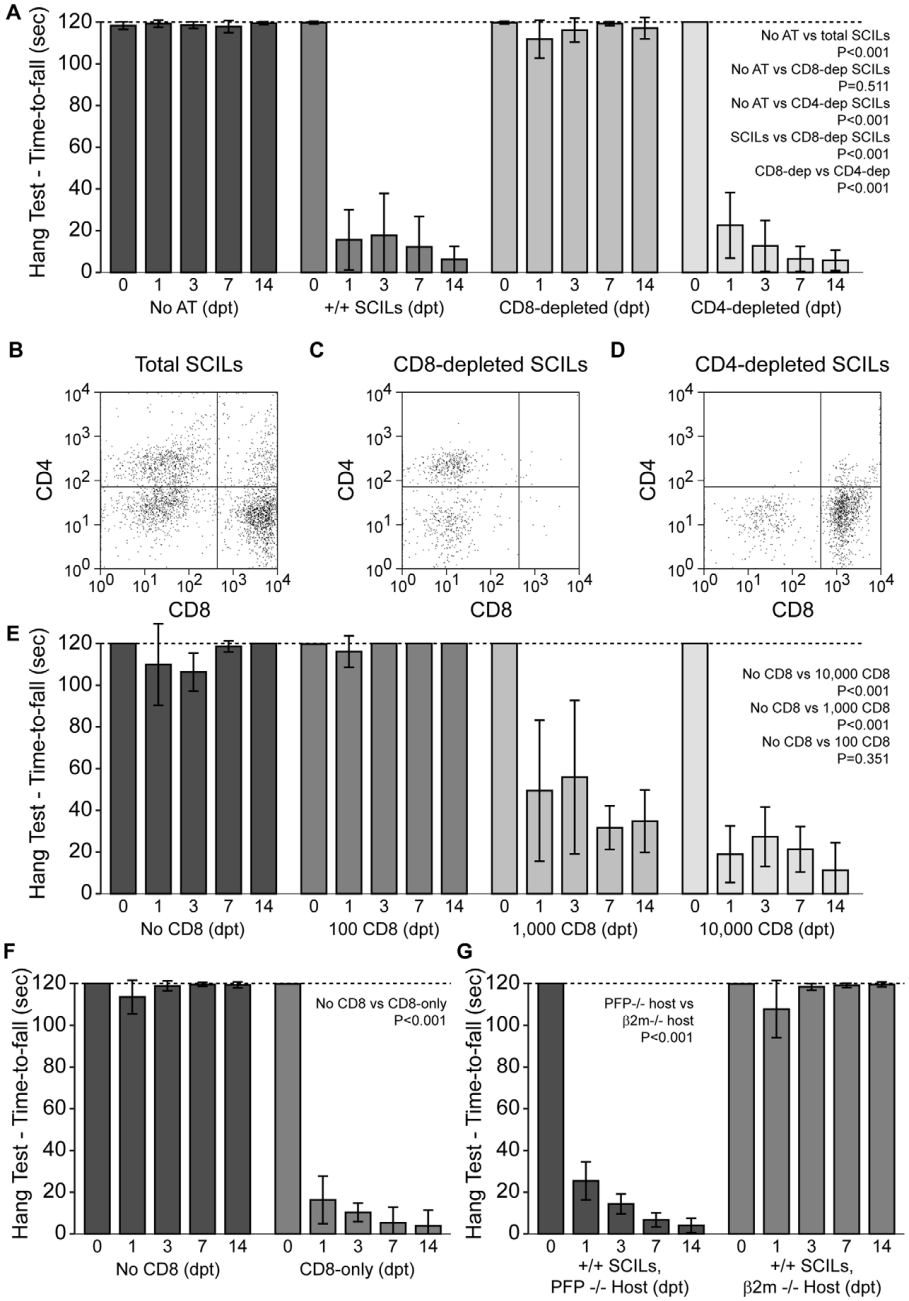
CD8^+^ T cells are necessary and sufficient for loss of function and axon injury following adoptive transfer. CD8^+^ spinal cord infiltrating T cells are necessary and sufficient to injure demyelinated axons in a perforin- and MHC class I-dependent manner. All data are presented as mean ±95% C.I. Statistical significance defined as *P<0.05, **P<0.01, ***P<0.001. Significance determined by two-way ANOVA with SNK pairwise posthoc analysis. All SCILs derived from 90 dpi perforin-competent donors. No CD8  =  CD8-depleted SCILs; CD8-only  =  immuno-isolated perforin-competent CD8^+^ SCILs; β2m−/−  =  β2 microglobulin-deficient B10Q host; CON  =  control IgG-treated; αCD8  =  anti-CD8 IgG-treated; PBMC  =  peripheral blood mononuclear cells. (A) Adoptive transfer of CD8-depleted SCILs did not induce loss of motor function, as compared to adoptive transfer of total SCILs or CD4-depleted SCILs. (B–D) Representative flow plots of total SCILs (B), CD8-depleted SCILs (C), and CD4-depleted SCILs (D) used for adoptive transfer. (E) Immuno-isolated CD8^+^ SCILS were added back to CD8-depleted total SCILs to determine how many were necessary to induce loss of motor function. Even 1,000 CD8^+^ SCILs were competent to significantly impair function. (F) Adoptive transfer of 10,000 immuno-isolated CD8^+^ SCILs, in the absence of any other cell type, induced a significant loss of function as compared to adoptive transfer of CD8-depleted SCILs. (G) Adoptive transfer of total perforin-competent SCILs into perforin-deficient hosts versus β2 microglobulin-deficient hosts revealed that surface expression of MHC class I is necessary for loss of motor function.

We next applied our adoptive transfer model to the identification of the primary effector leukocyte in the SCILs preparation. To do so, we prepared column-depleted perforin-competent SCILs preparations deficient in CD8^+^ cells, CD4^+^ cells, or NK1.1^+^ cells. In order to control for possible differences in total cell load, all depleted and non-depleted populations were adoptively transferred at 10^5^ cells per host. As before, we found that adoptive transfer of the total perforin-competent SCILs (Figure 6B) into demyelinated perforin-deficient hosts at 90 dpi resulted in a rapid and persistent loss of motor function (P<0.001 vs no transfer) (Figure 6A). Strikingly, perforin-competent SCILs depleted of CD8^+^ cells (Figure 6C) did not induce loss of function in demyelinated perforin-deficient hosts (P = 0.933 vs no transfer) (Figure 6A) (Movie S4). In contrast, CD4-depleted (P<0.001 vs no transfer) (Figure 6A, 6D) and NK1.1-depleted (P<0.001 vs no transfer) (not shown) SCILs triggered the same loss of motor function observed in the total SCILs adoptive transfer.

To confirm the specific role of CD8^+^ T cells in the loss of motor function, we prepared total perforin-competent SCILs depleted of CD8^+^ cells and then added back 0, 100, 1000, or 10000 column-purified (negative selection) perforin-competent CD8^+^ cells. Following adoptive transfer of these specific numbers of CD8^+^ cells within the context of 10^5^ other cell types in the SCILs, we found that even 1000 CD8^+^ cells were sufficient to permanently disrupt motor function in demyelinated perforin-deficient hosts (P<0.001 vs CD8-depleted SCILs) (Figure 6E). Moreover, adoptive transfer of 10000 column-purified (negative selection) perforin-competent CD8^+^ cells in the absence of any other cell type resulted in robust loss of motor function in demyelinated perforin-deficient hosts (P<0.001 vs 10^5^ CD8-depleted SCILs) (Figure 6F). Finally, this CD8^+^ T cell-mediated, perforin-dependent loss of motor function did not occur following adoptive transfer of SCILs into demyelinated β_2_microglobulin-deficient B10Q host mice (P<0.001 β_2_m−/− hosts vs perforin-deficient hosts) (Figure 6G). We conclude that the CD8^+^ T cell effect required the presence of surface MHC class I molecules within the nervous system.

### SCILs injure axons of cultured cortical neurons in a perforin-dependent manner

**Figure 7:**
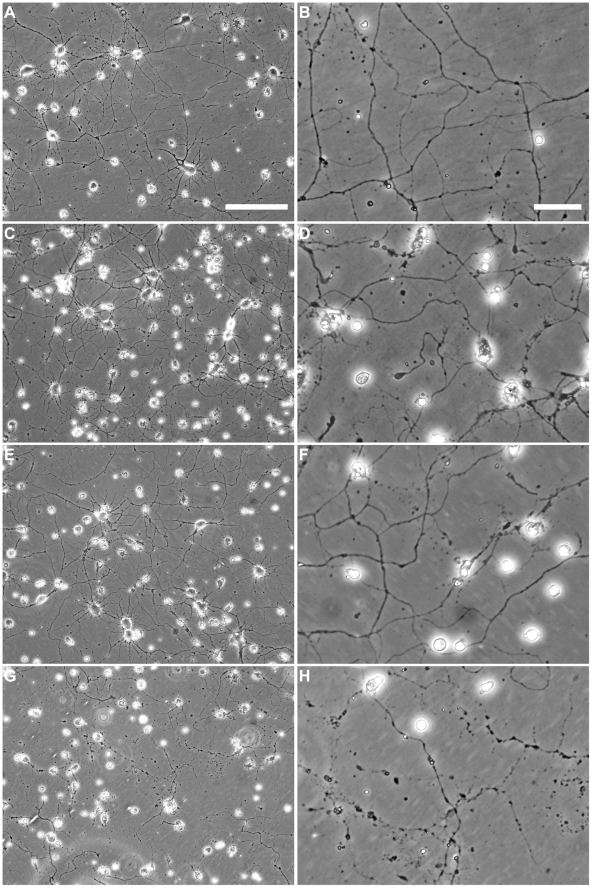
SCILs directly injure stressed neurites in vitro in the absence of viral infection. The neurites of cortical neurons non-lethally stressed by withdrawal of trophic factor support are targets of SCILs-mediated injury despite the complete absence of viral infection. (A, B) Trophic withdrawal alone is insufficient to injure cortical neuron neurites. (C, D) Addition of SCILs collected from 90 dpi B10.Q mice to non-stressed cortical neuron cultures for 120 min did not induce neurite injury. (E, F) Addition of SCILs to stressed cortical neurons for 15 min also did not result in any discernable neurite injury. (G, H) However, by 120 min of SCILs co-incubation with stressed cortical neurons, widespread neurite beading and disintegration was observed. Scale bar in (A) is 100 µm and refers to (A, C, E, G). Scale bar in (B) is 25 µm and refers to (B, D, F, H).

Evidence from our adoptive transfer model strongly suggested that CD8^+^ T cells in the SCILs are directly axopathic. In order to further test this hypothesis we asked whether SCILs can injure the axons of primary cortical neurons in culture. We have previously proposed that demyelination is a form of axonal stress associated with trophic factor withdrawal [5]. Based on this, we cultured H-2^q^ cortical neurons for one week in the presence of a growth factor cocktail containing BDNF and IGF-1. Withdrawal of BDNF from this cocktail for 24 hr triggered a stress response in the neurons that was marked by a variety of transcriptional responses, including upregulation of MHC class I (data not shown). Importantly, withdrawal alone was insufficient to cause apparent neurite injury (Figure 7A, 7B). Likewise, addition of SCILs collected from 90 dpi B10.Q mice to cortical neurons that did not experience trophic withdrawal stress did not induce any neurite damage after 120 min of co-incubation, despite the fact that the SCILs appeared to interact with the neurites (Figure 7C, 7D). In contrast, addition of SCILs to cortical neurons experiencing trophic factor withdrawal stress triggered widespread neurite injury after 120 min co-incubation (Figure 7G, 7H). This injury was marked by neurite beading and disintegration. Of note, the evolution of this neurite injury required prolonged interaction, as 15 min co-incubation of SCILs and stressed neurons was insufficient to trigger observable neurite injury (Figure 7E, 7F).

**Figure 8:**
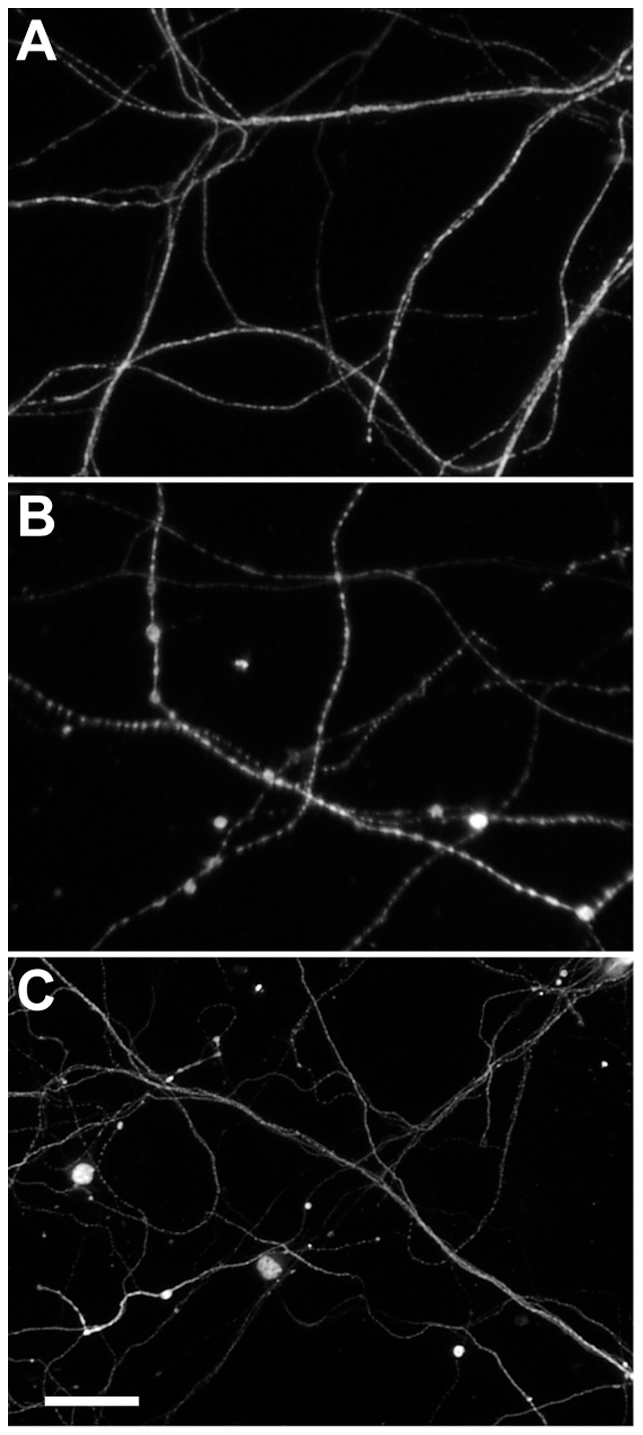
SCILs directly injure stressed axons in vitro in a perforin-dependent manner. Perforin-competent but not perforin-deficient SCILs induce beading and fragmentation of neurofilaments in cortical neuron axons in vitro in the absence of viral infection. (A) Neurofilament staining in cortical neuron axons not exposed to SCILs. (B) Beading and disintegration of neurofilaments elicited by perforin-competent SCILs incubated with cortical neurons for 120 min. (C) Perforin-deficient SCILs did not trigger axon injury. Scale bar in (C) is 50 µm and refers to (A–C).

Since lymphocytes and other cells present in the SCILs display a wide range of effector mechanisms we directly tested the role of perforin in the injury of axons in our in vitro system. As with our in vivo observations, we found that only perforin-competent SCILs were able to elicit axon injury as marked by beading and disintegration of neurofilaments (Figure 8B). The axons of cells incubated with perforin-deficient SCILs (Figure 8C) were indistinguishable from untreated axons (Figure 8A). We conclude that stressed axons are a direct target of perforin-mediated injury provoked by SCILs. Critically, because the cultured cortical neurons were not infected with TMEV, we conclude that the axopathic CD8^+^ T cells in the SCILs population are recognizing a neuron-derived epitope rather than a viral epitope on MHC class I. Further work is required to identify the relevant epitopes and the detailed mechanisms of perforin-mediated axon injury.

### Therapeutic immunodepletion of CD8^+^ T cells protects function in demyelinated mice

**Figure 9:**
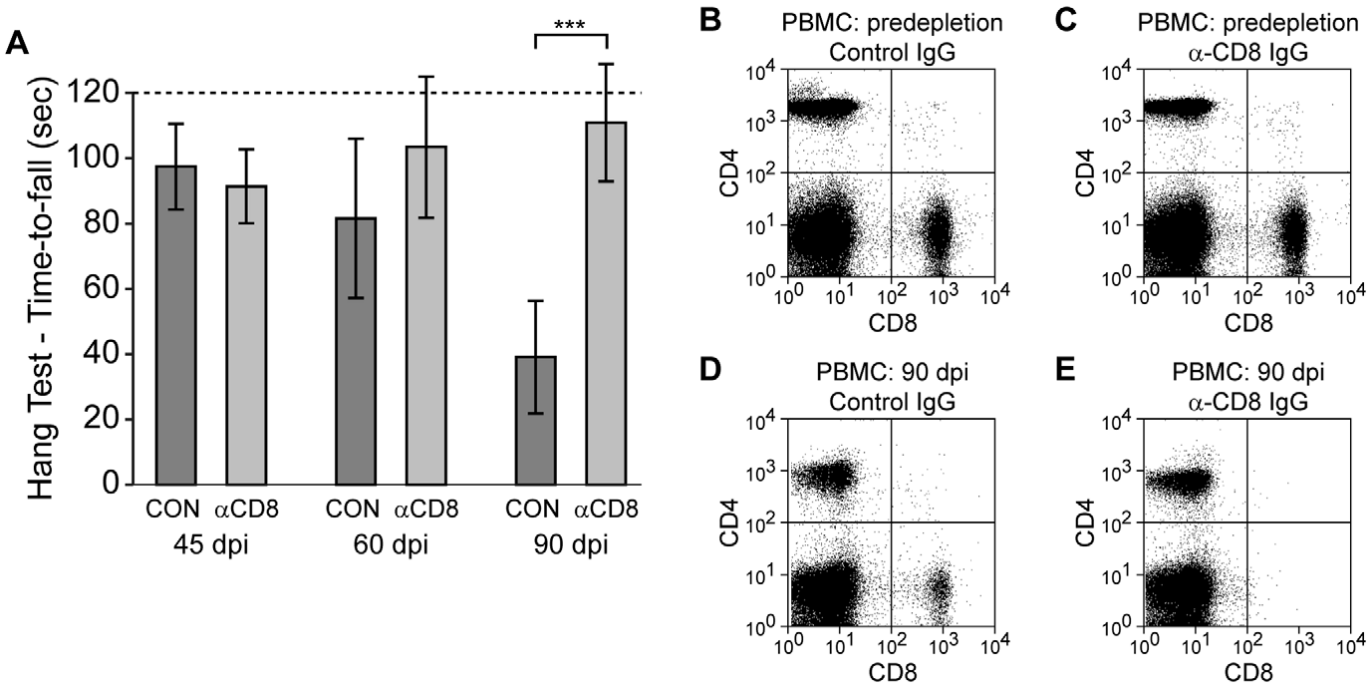
Depletion of CD8^+^ T cells preserves neurologic function. (A) Therapeutic immunodepletion of CD8^+^ T cells from 45 to 90 dpi results in preservation of motor function in chronically demyelinated B10Q mice. (B–E) Representative flow plots showing CD8-immunodepletion efficiency.

The robust role of CD8^+^ T cells in our SCILs adoptive transfer model suggested that therapeutic abrogation of these effectors in wild type B10Q mice during the course of axon injury would preserve motor function. We have previously observed a temporal disconnection between demyelination (21–45 dpi) and axon injury (>45 dpi) in chronically infected B10Q mice [19]. Therefore, we initiated immunodepletion of CD8^+^ T cells via weekly intraperitoneal injections of 0.5 mg of the 2.43 anti-CD8 clone from 45 dpi to 90 dpi. Depletion efficiency was assessed at 52, 75, and 90 dpi by flow cytometry of tail vein blood (Figure 9B–E) and compared to B10Q mice treated with a control Ig (OKM1). Motor function, as measured by hanging wire, did not differ between control and anti-CD8-treated mice at 60 dpi, but function was clearly protected in the CD8 immunodepleted mice at 90 dpi (P<0.001 control vs anti-CD8 at 90 dpi; P = 0.571 45 dpi anti-CD8 vs 90 dpi anti-CD8; P = 0.002 45 dpi control vs 90 dpi control) (Figure 9A). Despite the difference in function between the groups, viral load did not differ between control (5.7±1.2 log_10_ viral copies) and anti-CD8-treated mice (5.7±0.6 log_10_ viral copies) at 90 dpi (P = 0.948). Likewise, demyelinated lesion load did not differ between control (34%±14%) and anti-CD8-treated mice (35%±10%) (P = 0.880). We conclude that immunodepletion of CD8^+^ T cells may be a valuable tool for preventing axon injury in the demyelinated central nervous system.

## Discussion

Keeping in mind the pathogenic, mechanistic, and pathological heterogeneity of MS, our findings suggest that CD8^+^ T cells, working via a perforin-dependent mechanism, are the primary effector population responsible for injury and loss of demyelinated axons. Although perforin-dependent CD8^+^ T cells are both necessary and sufficient to injure demyelinated axons and destroy motor function, this effector mechanism is irrelevant to demyelination in H-2^q^ MHC haplotype mice chronically infected with TMEV. Indeed, in the absence of perforin, the preservation of motor function to uninfected levels despite the presence of considerable spinal cord demyelination is reminiscent of a radiologically isolated demyelinating syndrome [25]. While our adoptive transfer experiments do not address the relevance of non-perforin CD8^+^ T cell effector mechanisms to demyelination, our observation that therapeutic depletion of CD8^+^ T cells during the chronic phase of disease protects motor function without altering demyelination argues that these cells are predominantly involved in direct injury of axons. Our findings also lend strong support to the idea that demyelination is necessary, but not sufficient, to damage axons and chronically disrupt motor function.

Although we have only just begun to explore the role of MHC class I in the injury of axons in our model, several observations suggest that class I expression is necessary for CD8^+^ T cell-mediated damage. First, adoptive transfer of perforin-competent CD8^+^ T cells collected from chronically demyelinated wildtype H-2^q^ mice into demyelinated but functionally preserved β_2_-microglobulin deficient H-2^q^ mice did not result in loss of motor function (Figure 6G). This suggests that surface expression of MHC class I in the donor mice is required for CD8^+^ T cell recognition of demyelinated axons. Second, we have found that adoptive transfer of perforin-competent CD8^+^ T cells collected from chronically demyelinated wild type H-2^q^ mice into demyelinated but functionally preserved perforin-deficient H-2^b^ mice [26] also does not result in loss of function, suggesting that MHC mismatch prevents CD8^+^ T cell recognition of the demyelinated axons (data not shown). Third, in preliminary experiments we have found that CD8^+^ SCILs recognize and injure axons of cultured H-2^q^ cortical neurons (Figures 7 and 8) but do not recognize or injure cultured H-2^b^ cortical neurons (data not shown). Fourth, we have observed MHC class I immunostaining on longitudinally transverse fiber-like structures in demyelinated lesions and these structures exhibit markers of axon injury and are in proximity to CD8^+^ T cells (Figures 2 and 3).

Despite the apparent MHC class I dependence of CD8+ T cell-mediated axon injury, our observations suggest that these T cells are not antiviral. While we have started exploring possible autoantigens presented by MHC class I on demyelinated axons, our current findings only suggest that viral epitopes are not relevant. We have recently published an extensive characterization of CD8^+^ T cells present in the spinal cord at 90 dpi and concluded that these cells, although evidently activated acute effectors, were not competent to kill virus-infected targets in vitro [23]. Likewise, in our current experiments, adoptive transfer of large numbers of perforin-competent antiviral splenocytes into demyelinated but functionally preserved perforin-deficient hosts failed to impact motor function, suggesting that antiviral reactivity is not sufficient to explain axon injury. In addition, perforin-competent SCILs robustly damaged the neurites of cultured cortical neurons that were never infected (Figures 7 and 8), suggesting that antiviral reactivity is not necessary for axon injury. Especially with regard to CD8^+^ T cell-mediated injury of demyelinated corticospinal motor axons, this virus independence is consistent with the absence of chronic infection in the brain at 90 dpi [19]. In ongoing experiments using non-viral models of demyelination we intend to directly determine whether CD8^+^ T cells isolated from the demyelinated spinal cord at 90 dpi are competent to damage demyelinated axons in uninfected hosts.

We recognize that CD8^+^ T cells may play a role via other effector mechanisms in myelin damage [27], immune regulation [28], or even in myelin repair [29]. We also acknowledge that the role of CD8^+^ T cells in axon injury may be complicated or reversed in some animal models of demyelinating disease [30]. However, our current findings provoke a cautious optimism that manipulation of CD8^+^ T cells may be therapeutically relevant to human MS. While the field has largely concentrated on therapeutic intervention in the function of CD4^+^ T cells in CNS, we suggest that explicitly targeting CD8^+^ T cells may be a viable option for preserving axons. A CD8^+^ T cell depleting or blocking therapy may permit considerable stabilization of disease progression, essentially preventing the conversion of relapsing-remitting patients to a secondary progrssive phase. At the same time, a CD8-centric therapy may sidestep the complications of immunocompromise associated with therapeutic blockade of CNS infiltration by all lymphocytes [31].

## Materials and Methods

Detailed methods are provided in File S1.

### Ethics Statement

Animal use and care were in accordance with National Institutes of Health guidelines. All procedures were approved by the Mayo Clinic Institutional Animal Care and Use Committee (NIH OLAW assurance number A3291-01).

### Mice

Perforin-deficient mice on an H-2^q^ background were previously described [19]. Homozygous beta-2 microglobulin (β2m)-deficient mice on an H-2^b^ background were obtained from the Whitehead Institute and were subsequently bred on an H-2^q^ background in our facility. Gender was mixed for all experimental groups.

### Virus

The Daniel's strain of TMEV was used for all experiments. At 4–6 weeks of age, mice were inoculated intracerebrally with 2×10^5^ PFU of TMEV in a total volume of 10 µL.

### Preparation of cells and adoptive transfer

Spinal cord infiltrating leukocytes (SCILs), peripheral blood mononuclear cells, and splenocytes were prepared as previously described [23]. SCILs or other cell populations were resuspended in RPMI to a final concentration of 10^6^ cells per mL. Each mouse received 100 µL of cell suspension via tail vein injection.

### Histopathology of spinal cord tissue and axon analysis

Demyelination was measured by camera lucida drawing of 4% *para*-phenylenediamine-stained sections as previously described [19], [21], [24]. Axons at the T6 thoracic level were automatically analyzed at 60X magnification from each animal according to a sampling scheme previously developed [21]. Images were captured from regions containing no demyelination to ensure the measurement of only myelinated fibers. Each field measured 17,675 µm^2^. A total of 2.16 mm^2^ from sham-infected controls, 1.76 mm^2^ from perforin-competent mice, and 1.34 mm^2^ from perforin-deficient mice were collected at 90 dpi. A total of 0.74 mm^2^ from perforin-deficient hosts receiving sham adoptive transfer, 0.53 mm^2^ from perforin-deficient hosts receiving adoptive transfer of SCILs from perforin-competent donors, and 0.73 mm^2^ from perforin-deficient hosts receiving adoptive transfer of SCILs from perforin-deficient donors were collected.

### Behavioral analyses and electrophysiology

Behavioral measurements and electrophysiology methodology are described in detail in the supplemental methods.

### In vivo immunodepletion of CD8^+^ T cells

Wildtype B10Q mice were immunodepleted of CD8^+^ T cells via weekly intraperitoneal injections of 0.5 mg of the anti-CD8 2.43 clone (ATCC, No. TIB 210) from 45 dpi to 90 dpi. Control animals were treated in parallel with 0.5 mg of the control antibody OKM1 (ATCC, No. CRL 8026). Antibodies were prepared by ammonium sulfate precipitation from 2.43 and OKM1 hybridoma supernatants.

### Statistics

All data are presented as mean±95% confidence interval (CI). Statistical tests for every analysis are described in detail in Table S1. Normality was used when appropriate to determine whether parametric or non-parametric tests were employed. All calculations were performed using SigmaPlot 11 (Systat Software, Chicago, IL). Every experiment was repeated a minimum of 3 times on separate animal cohorts.

## Supporting Information

Figure S1 Absolute numbers of CD45^hi^CD8^+^ T cells and CD45^hi^CD4^+^ T cells do not differ in SCILs prepared from perforin competent and perforin deficient hosts at 90 dpi. No differences in the percentages of CD8^+^ cells and CD4^+^ cells (expressed as a percentage of CD45^hi^ T cells) are seen in SCILs prepared from perforin competent versus perforin deficient hosts at 90 dpi. All data are expressed as mean ±95% C.I. Significance was analyzed by t-test.
(0.10 MB TIF)

Figure S2 Motor function as assessed by direct observation of posture is impaired in perforin competent hosts (a), but is preserved in perforin deficient hosts (b).
(1.75 MB TIF)

File S1 Additional materials and methods.
(0.06 MB DOC)

Movie S1 Open field video analysis demonstrates preservation of motor function in perforin deficient mice and impairment of motor function in perforin competent mice at 90 dpi.
(5.60 MB MOV)

Movie S2 Runway video analysis demonstrates preservation of motor function in perforin deficient mice and impairment of motor function in perforin competent mice at 90 dpi.
(3.53 MB MOV)

Movie S3 Runway video analysis demonstrates loss of motor function in perforin deficient hosts after adoptive transfer of SCILs from perforin competent donors.
(3.81 MB MOV)

Movie S4 Open field video analysis demonstrates motor impairment in perforin deficient hosts after adoptive transfer of total SCILs from perforin competent donors, whereas no impairment occurs in perforin deficient hosts after adoptive transfer of CD8^+^ depleted SCILs from perforin competent donors.
(5.20 MB MOV)

## References

[1] Weiner HL (2009). The challenge of multiple sclerosis: how do we cure a chronic heterogeneous disease?. Ann Neurol.

[2] Friese MA, Fugger L (2005). Autoreactive CD8+ T cells in multiple sclerosis: a new target for therapy?. Brain.

[3] Friese MA, Fugger L (2009). Pathogenic CD8(+) T cells in multiple sclerosis.. Ann Neurol.

[4] Goverman J, Perchellet A, Huseby ES (2005). The role of CD8(+) T cells in multiple sclerosis and its animal models.. Curr Drug Targets Inflamm Allergy.

[5] Howe CL (2008). Immunological aspects of axon injury in multiple sclerosis.. Curr Top Microbiol Immunol.

[6] Babbe H, Roers A, Waisman A, Lassmann H, Goebels N (2000). Clonal expansions of CD8(+) T cells dominate the T cell infiltrate in active multiple sclerosis lesions as shown by micromanipulation and single cell polymerase chain reaction.. J Exp Med.

[7] Hauser SL, Bhan AK, Gilles F, Kemp M, Kerr C (1986). Immunohistochemical analysis of the cellular infiltrate in multiple sclerosis lesions.. Ann Neurol.

[8] Kuhlmann T, Lingfeld G, Bitsch A, Schuchardt J, Bruck W (2002). Acute axonal damage in multiple sclerosis is most extensive in early disease stages and decreases over time.. Brain.

[9] Jacobsen M, Cepok S, Quak E, Happel M, Gaber R (2002). Oligoclonal expansion of memory CD8+ T cells in cerebrospinal fluid from multiple sclerosis patients.. Brain.

[10] Skulina C, Schmidt S, Dornmair K, Babbe H, Roers A (2004). Multiple sclerosis: brain-infiltrating CD8+ T cells persist as clonal expansions in the cerebrospinal fluid and blood.. Proc Natl Acad Sci U S A.

[11] Junker A, Ivanidze J, Malotka J, Eiglmeier I, Lassmann H (2007). Multiple sclerosis: T-cell receptor expression in distinct brain regions.. Brain.

[12] Hoftberger R, Aboul-Enein F, Brueck W, Lucchinetti C, Rodriguez M (2004). Expression of major histocompatibility complex class I molecules on the different cell types in multiple sclerosis lesions.. Brain Pathol.

[13] van Oosten BW, Lai M, Hodgkinson S, Barkhof F, Miller DH (1997). Treatment of multiple sclerosis with the monoclonal anti-CD4 antibody cM-T412: results of a randomized, double-blind, placebo-controlled, MR-monitored phase II trial.. Neurology.

[14] Coles AJ, Compston DA, Selmaj KW, Lake SL, Moran S (2008). Alemtuzumab vs. interferon beta-1a in early multiple sclerosis.. N Engl J Med.

[15] Polman CH, O'Connor PW, Havrdova E, Hutchinson M, Kappos L (2006). A randomized, placebo-controlled trial of natalizumab for relapsing multiple sclerosis.. N Engl J Med.

[16] Havrdova E, Galetta S, Hutchinson M, Stefoski D, Bates D (2009). Effect of natalizumab on clinical and radiological disease activity in multiple sclerosis: a retrospective analysis of the Natalizumab Safety and Efficacy in Relapsing-Remitting Multiple Sclerosis (AFFIRM) study.. Lancet Neurol.

[17] Goverman J (2009). Autoimmune T cell responses in the central nervous system.. Nat Rev Immunol.

[18] Malmestrom C, Lycke J, Haghighi S, Andersen O, Carlsson L (2008). Relapses in multiple sclerosis are associated with increased CD8+ T-cell mediated cytotoxicity in CSF.. J Neuroimmunol.

[19] Deb C, Lafrance-Corey RG, Zoecklein L, Papke L, Rodriguez M (2009). Demyelinated axons and motor function are protected by genetic deletion of perforin in a mouse model of multiple sclerosis.. J Neuropathol Exp Neurol.

[20] Howe CL, Ure D, Adelson JD, Lafrance-Corey R, Johnson A (2007). CD8+ T cells directed against a viral peptide contribute to loss of motor function by disrupting axonal transport in a viral model of fulminant demyelination.. J Neuroimmunol.

[21] Howe CL, Adelson JD, Rodriguez M (2007). Absence of perforin expression confers axonal protection despite demyelination.. Neurobiol Dis.

[22] Murray PD, McGavern DB, Lin X, Njenga MK, Leibowitz J (1998). Perforin-dependent neurologic injury in a viral model of multiple sclerosis.. J Neurosci.

[23] Deb C, Howe CL (2009). Functional characterization of mouse spinal cord infiltrating CD8+ lymphocytes.. J Neuroimmunol.

[24] Rodriguez M (1991). Immunoglobulins stimulate central nervous system remyelination: electron microscopic and morphometric analysis of proliferating cells.. Lab Invest.

[25] Okuda DT (2009). Unanticipated demyelinating pathology of the CNS.. Nat Rev Neurol.

[26] Rivera-Quinones C, McGavern D, Schmelzer JD, Hunter SF, Low PA (1998). Absence of neurological deficits following extensive demyelination in a class I-deficient murine model of multiple sclerosis.. Nat Med.

[27] Sobottka B, Harrer MD, Ziegler U, Fischer K, Wiendl H (2009). Collateral Bystander Damage by Myelin-Directed CD8+ T Cells Causes Axonal Loss.. Am J Pathol.

[28] Chen ML, Yan BS, Kozoriz D, Weiner HL (2009). Novel CD8(+) Treg suppress EAE by TGF-beta- and IFN-gamma-dependent mechanisms.. Eur J Immunol.

[29] Bieber AJ, Kerr S, Rodriguez M (2003). Efficient central nervous system remyelination requires T cells.. Ann Neurol.

[30] Begolka WS, Haynes LM, Olson JK, Padilla J, Neville KL (2001). CD8-deficient SJL mice display enhanced susceptibility to Theiler's virus infection and increased demyelinating pathology.. J Neurovirol.

[31] Linda H, von Heijne A, Major EO, Ryschkewitsch C, Berg J (2009). Progressive multifocal leukoencephalopathy after natalizumab monotherapy.. N Engl J Med.

