# Supplementary figures and images for: CD8+ T Cells Cause Disability and Axon Loss in a Mouse Model of Multiple Sclerosis
Source: PLoS One. 2010 Aug 30;5(8):e12478. doi: 10.1371/journal.pone.0012478 (PMC2930011; doi:10.1371/journal.pone.0012478)

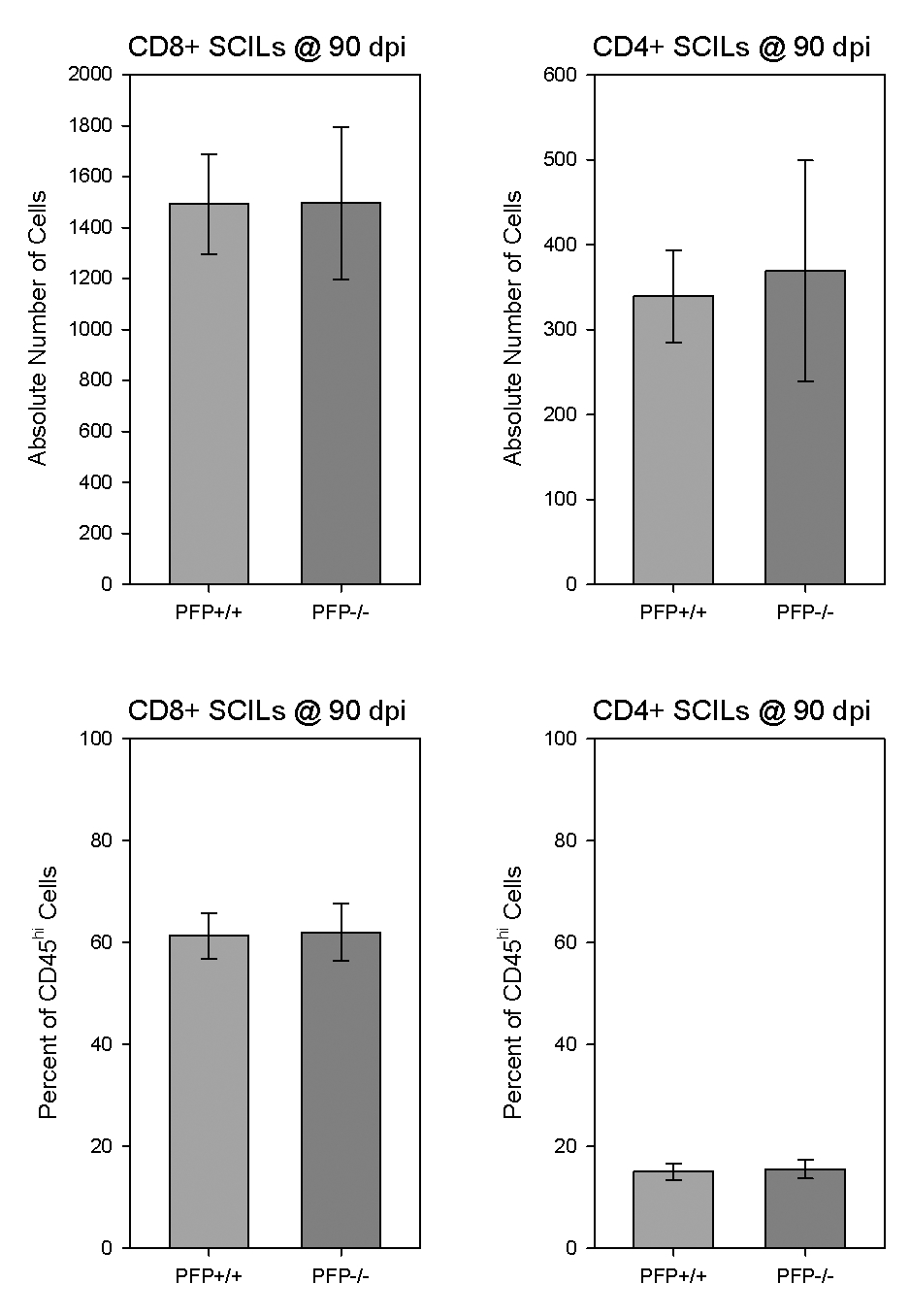

Supplement: Figure S1 — Absolute numbers of CD45hiCD8+ T cells and CD45hiCD4+ T cells do not differ in SCILs prepared from perforin competent and perforin deficient hosts at 90 dpi. No differences in the percentages of CD8+ cells and CD4+ cells (expressed as a percentage of CD45hi T cells) are seen in SCILs prepared from perforin competent versus perforin deficient hosts at 90 dpi. All data are expressed as mean ±95% C.I. Significance was analyzed by t-test. (0.10 MB TIF) [file pone.0012478.s001.tif]

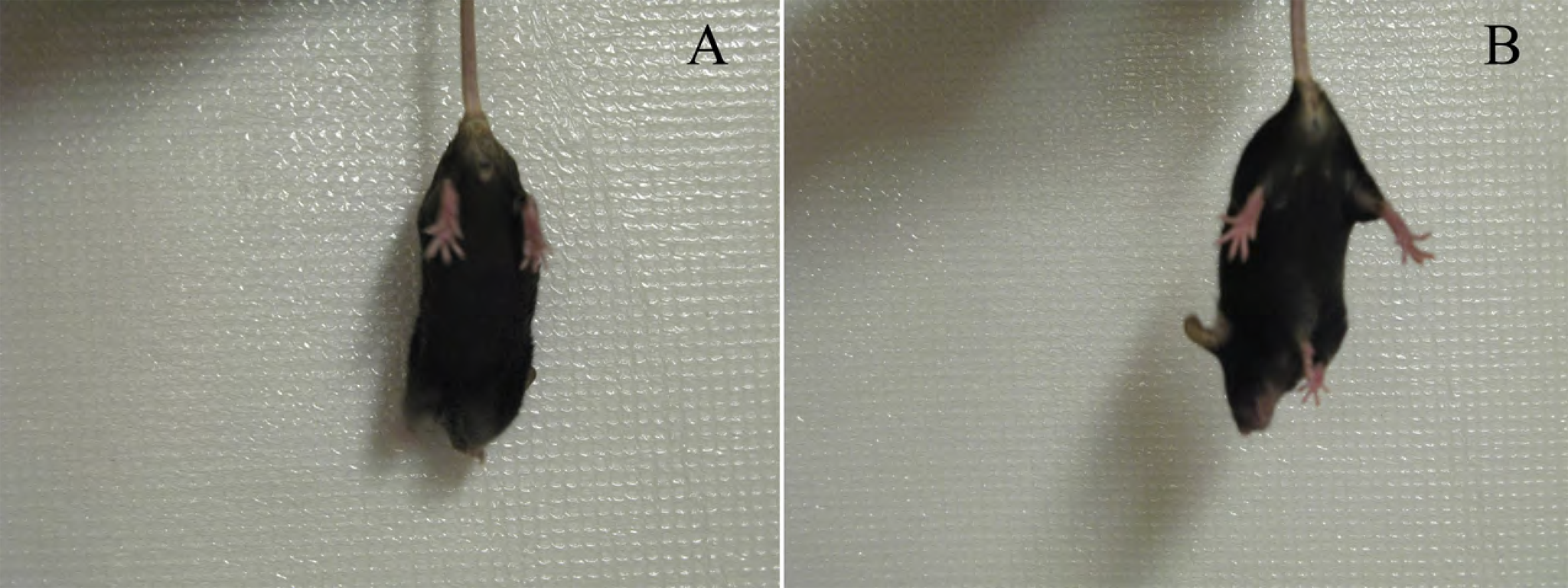

Supplement: Figure S2 — Motor function as assessed by direct observation of posture is impaired in perforin competent hosts (a), but is preserved in perforin deficient hosts (b). (1.75 MB TIF) [file pone.0012478.s002.tif]
