# Supplementary material for: CD8+ T Cells Cause Disability and Axon Loss in a Mouse Model of Multiple Sclerosis
Source: PLoS One. 2010 Aug 30;5(8):e12478. doi: 10.1371/journal.pone.0012478 (PMC2930011; doi:10.1371/journal.pone.0012478)
Supplement: File S1 — Additional materials and methods. (0.06 MB DOC) [file pone.0012478.s003.doc]

**Materials and Methods**

*Mice.*

Wildtype, perforin-competent B10.D1-H2^q^ (B10Q) mice were obtained from Jackson Laboratories (Bar Harbor, ME; No. 002024) and from subsequent breeding at our facility. C57Bl/6-Pfp^tm1sdz^ mice (Jackson Laboratories; No. 002407 ) are MHC class I H-2^b^ haplotype and have a targeted disruption of the perforin gene. Perforin-deficient mice on an H-2^q^ background were generated by crossing perforin knockout H-2^b^ mice to B10Q mice. F_1_ mice from this cross were mated to produce F_2_ breeding pairs, which were screened by PCR for the presence of the targeted disruption of the perforin allele. Mice were further screened by flow cytometry using antibodies to H-2^b^ and H‑2^q^. To establish a homozygous H-2^q^ line, only mice that were negative for H-2^b^ by flow cytometry were used for further breeding. These mice were subsequently backcrossed for more than ten generations with screening of offspring by both assays to confirm the homozygous H‑2^q^ perforin-deficient genotype. Homozygous beta-2 microglobulin (β2m)-deficient mice on an H-2^b^ background were originally obtained from the Whitehead Institute and were subsequently bred in our facility. Homozygous deletion of the β2m gene was confirmed by the absence of CD8^+^ T cells in the peripheral blood mononuclear cell population. β2m-deficient mice on an H-2^q^ background were generated by crossing β2m-deficient H‑2^b^ mice to B10Q mice. F_1_ mice from this cross were mated to produce F_2_ breeding pairs, which were screened by flow cytometry for the absence of CD8^+^ cells. Mice were further screened by flow cytometry using antibodies to H-2^b^ and H-2^q^. The homozygous H-2^q^ line was subsequently backcrossed for more than ten generations. Gender was mixed for all experimental groups. Animal use and care were in accordance with Mayo Clinic Institutional Animal Care and Use Committee and National Institutes of Health guidelines.

*Virus.*

The Daniel’s strain of TMEV was used for all experiments. At 4-6 weeks of age, mice were inoculated intracerebrally with 2 x 10^5^ PFU of TMEV in a total volume of 10 µL.

*Preparation of spinal cord infiltrating leukocytes (SCILs).*

At 90 days post infection (dpi), mice were euthanized with isoflurane overdose. Spinal columns were excised and the spinal cord was removed by insufflation using 20 mL of RPMI at 4°C expressed through a 19-gauge needle. The spinal cord was transferred into 10 mL of fresh, cold RPMI and homogenized in a Tenbroeck tissue grinder. The resulting cell suspension was gravity sieved through a 40 µM strainer (BD Biosciences, San Jose, CA, No. 352340) to remove large aggregates. Cells were pelleted by centrifugation at 800 g_ave_ for 5 minutes at 4°C and the supernatant was discarded. Cells were then resuspended in 1 mL of 70% isotonic Percoll (GE Healthcare, Piscataway, NJ, No. 17-0891-02) prepared in PBS, transferred to a 5 mL polystyrene round bottom tube and overlaid with 1 mL of 35% Percoll prepared in PBS. This gradient was centrifuged at 600 g_ave_ for 20 minutes at 4°C with braking disengaged to prevent disruption of the gradient interface layer. Myelin and other debris formed an opaque layer at the top of the gradient, while the cells of interest formed a layer at the interface between 35% and 70% Percoll. Following aspiration of myelin debris, the central interface was collected into 3 mL of fresh, cold RPMI. The cell suspension was pelleted by centrifugation at 1200 g_ave_ for 5 minutes at 4°C and the supernatant was discarded. The pellet was then processed for flow cytometry or adoptive transfer.

*Collection of peripheral blood mononuclear cells.*

Peripheral blood was collected by intracardiac puncture into heparinized tubes and diluted to 1.5 mL final volume with PBS. The diluted blood was layered over 1 mL of Ficoll-Paque Plus (GE Healthcare No. 17-1440-02) and centrifuged at 1200 g_ave_ for 25 minutes at 25°C with braking disengaged. PBMCs were collected at the interface, resuspended in PBS, and washed twice by centrifugation at 800 g_ave_ for 5 minutes at 25°C. The cell pellet was resuspended in PBS for analysis.

*Preparation of splenocytes.*

Spleens were collected in 1 mL RPMI and dissociated via blunt grinding. The tissue suspension was collected into a total volume of 2 mL RPMI and then gravity strained through a 40 µm cell strainer. This suspension was centrifuged at 900 g_ave_ for 5 minutes at 4°C, resuspended in RPMI, and delivered to host mice via tail vein injection.

*Preparation of lymphokine-activated killer cells.*

Splenocytes were collected as described above and then incubated for 4 days in medium (RPMI + 10% fetal bovine serum) containing IL-2 (Peprotech, Rocky Hill, NJ, No. 212-12) at a concentration of 1 µg/mL prior to adoptive transfer via tail vein injection.

*Adoptive Transfer*

SCILs or other cell populations were resuspended in RPMI to a final concentration of 10^6^ cells per mL. Each mouse received 100 μL of cell suspension via tail vein injection.

*Magnetic column depletion of CD4^+^, CD8^+^ and NK1.1^+^ cells.*

The SCILs suspension was passed through a separation filter (Miltenyi Biotec, Auburn, CA, No. 130-041-407) and flushed with 2 mL of MACS buffer consisting of MACS BSA Stock Solution (Miltenyi Biotec, Auburn, CA, No. 130-091-376) diluted 1:20 in autoMACS Rinsing Solution (Miltenyi No. 130-091-222). The eluate was then centrifuged at 300 g_ave_ for 10 minutes at 4°C. The cell pellet was resuspended in 90 µL of MACS buffer. Ten µL of FcR Blocking Reagent (Miltenyi, No. 130-092-575) was added and the mixture was incubated on ice for 10 minutes. Ten µL of the appropriate magnetic bead tagged antibody (NK1.1^+^ depletion: CD49b MicroBeads, Miltentyi, No. 130-052-501; CD4^+^ depletion: CD4 MicroBeads, Miltenyi, No. 130-049-201; CD8^+^ depletion: CD8 MicroBeads, Miltenyi, No. 130-049-401) was added to the mixture and incubated on ice for 15 minutes. Two mL of MACS buffer was added and the mixture was spun at 300 g_ave_ for ten minutes at 4°C . Cell pellets were resuspended in 500 µL MACS buffer. LD Columns (Miltenyi No. 130-042-901) were placed in a QuadroMACS separation unit (Miltenyi 130-091-051) and flushed with 2 mL of MACS buffer. Cell suspensions were then added to the column and the column was washed twice with 1 mL of MACS buffer. The depleted eluate was then spun at 500 g_ave_ for 10 minutes at 4°C and the cell pellet was resuspended in RPMI for adoptive transfer.

*Flow cytometry.*

All experiments represent analysis of SCILs prepared from individual animals. Cells were resuspended in PBS containing 1% fetal bovine serum and 0.02% sodium azide. The cell suspension was incubated with blocking buffer (50% (v/v) supernatant from 2.4G2 hybridoma [Fc block; anti-CD16/32; ATCC, Manassas, VA, No. HB-197]) for 15 minutes at 4°C. Primary antibodies against extracellular antigens were added to the blocked cells at 1:100 and incubated for 30 minutes at 4°C. CD45 was detected with clone 30-F11 (BD Biosciences No. 553080). CD8 was detected with clone 53-6.7 (BD Biosciences No. 553035). CD4 was detected with clone GK1.5 (BD Biosciences No. 553730). Stained cells were washed once with PBS containing 1% fetal bovine serum and 0.02% sodium azide and then fixed in 2% paraformaldehyde for 30 minutes prior to flow cytometric analysis on a BD FACSCalibur. FCS files were analyzed offline using FlowJo 7.2 (Windows version; Tree Star, Inc., Ashland, OR).

*Histopathology of spinal cord tissue.*

Mice were euthanized with sodium pentobarbital overdose and perfused with 50 mL of Trump’s fixative via intracardiac puncture. Intact spinal columns were removed and post-fixed for 24-48 hours in Trump’s fixative. The spinal cord was manually dissected from the spinal column and cut into one mm coronal blocks. Every third block was osmicated and embedded in glycol methacrylate [1]. Sections were stained for 20 minutes with 4% *para*-phenylenediamine to reveal myelin. Total white matter and demyelinated lesions were traced by hand using a camera lucida system, followed by digitization and calculation of areas using Image J software. Measurements were made on 10 spinal cord sections from each animal. Percent demyelination was calculated by dividing the lesion area by the total white matter area and multiplying by 100.

*Axon analysis.*

Sections stained with 4% para-phenylenediamine were collected from the smallest thoracic spinal cord section (T6). Digitized images were collected at 60X magnification from each animal according to a sampling scheme previously developed [2]. Images were captured from regions containing no demyelination to ensure the measurement of only myelinated fibers. Each field measured 17,675 µm^2^. A total of 2.16 mm^2^ from sham-infected controls, 1.76 mm^2^ from perforin-competent mice, and 1.34 mm^2^ from perforin-deficient mice were collected at 90 dpi. A total of 0.74 mm^2^ from perforin-deficient hosts receiving sham adoptive transfer, 0.53 mm^2^ from perforin-deficient hosts receiving adoptive transfer of SCILs from perforin-competent donors, and 0.73 mm^2^ from perforin-deficient hosts receiving adoptive transfer of SCILs from perforin-deficient donors were collected. Myelinated axon diameters were calculated after segmentation of the gray values corresponding to the axoplasm from each image. Batch algorithms were generated in MatLab (The Mathworks, Natick, MA) to automatically calculate the diameter of each axon in the field from the segmented binary image after regions corresponding to the vasculature, cell bodies, longitudinal axons, and demyelination were excluded on the basis of circularity thresholding. Diameters less than 0.5 µm were excluded from analysis to eliminate small regions that did not correspond to axons. Axon area measurements were binned for analysis: small (1-4 µm^2^); medium (4-10 µm^2^); large (>10 µm^2^). The number of axons in each bin was normalized to the area of spinal cord analyzed to yield axons per mm^2^. These values were averaged across all animals per group.

*Viral load.*

The VP2 fragment of TMEV was amplified by RT-PCR with gene-specific primers from total RNA. Glyceraldehyde-3-phosphate dehydrogenase (GAPDH) was used as a control for intersample variability. Primers and reaction conditions were as previously described [3]. The accumulation of product was monitored by SYBR green fluorescence at the completion of each cycle. There was a direct relationship between the cycle number at which the accumulation of PCR products became exponential and the log concentration of RNA molecules initially present. The amount of viral RNA was expressed as log_10_ virus copy number per µg total RNA.

*Rotarod.*

An accelerating Rotamex rotarod (Columbus Instruments, Columbus, OH) was used to assess motor function as previously described [2,4]. Mice were exposed to a 4 day training protocol prior to TMEV infection or sham infection. The training protocol consisted of three trials per day at the following speeds with each trial separated by 10-15 minutes: Day 1, constant 10 rpm for 3 minutes; Day 2, accelerating from 10-20 rpm for 3 minutes; Day 3, accelerating from 10-30 rpm for 3 minutes; Day 4, accelerating from 10-40 rpm for 3 minutes. At the conclusion of initial training, animals were tested using three trials in which the rod accelerated from 10 to 70 rpm over 6 minutes. At subsequent time points, animals were re-trained for three trials with the rod accelerating from 10 to 40 rpm over 3 minutes on the day prior to testing. On the next day, experimental testing was performed as described above. Data are expressed as mean time-to-fall from the rotarod.

*Hanging wire.*

Mice were placed at the midpoint of a stainless steel cage lid (29 cm by 18 cm with 2 mm diameter wires). The apparatus was inverted and animals were observed until time to fall, up to a maximum of 120 seconds. The number of seconds spent hanging was recorded as time-to-fall. All animals were subjected to two consecutive trials with at least a 10 minute interval between trials [5].

*Footprint analysis.*

Prior to footprint analysis, mice were acclimated by allowing two consecutive runs down an enclosed acrylic walkway with a darkened box placed at the end of the walkway. Subsequently, the fore-and hindlimb paws were painted with non-toxic red and blue paint (RoseArt Industries, Livingston, NJ). Mice were placed at the start of the walkway and allowed to walk along a strip of white paper to the escape chamber. Prints were digitized using a color scanner. A section of the image containing three consecutive, clearly identifiable prints from each limb was identified and the stride-to-stride lengths between prints from each limb were measured. The aggregate stride length is reported as the mean of all of the individual stride-to-stride lengths for a given mouse.

*Electrophysiology*

Electrophysiology was performed using a Nicolet Viking IV (VIASYS Healthcare). Mice were anesthetized by ketamine and xylazine; body temperature was maintained at 35°C by heating pad with realtime feedback. The scalp and lower left leg of the mice were shaved and the stimulation sites were marked in black ink on the skin. We stimulated at two different sites. For the first, the cathode tip was inserted through the scalp to contact the periosteum of the bregmatic suture at the intersection of the midline and the interaural line and the anode tip was placed 3-4 mm lateral and anterior to the cathode, in contact with the temporal bone just anterior to the ear. At the second site, a small posterior midline incision was made at the junction between the brainstem and high cervical spinal cord and the cathode and anode were inserted in contact with the periosteum to provide stimulation across the cord. Two disposable subdermal stainless steel EEG needles (27G, 12 mm long) were used as electrodes to excite the underlying motor cortex or the high cervical cord. Paired stimulation with 2 ms interstimulus interval was used and the intensity was determined by measuring the resting motor threshold (RMT), defined as the lowest current intensity (mA) required to get 3 responses of greater than 50 μV from 6 consecutive stimuli. For all mice, stimulus intensity was set to 150% of RMT throughout the experiment. To record motor evoked potentials (MEPs) an active electrode was placed subcutaneously over the gastrocnemius muscle, a reference electrode was inserted next to the Achilles tendon, and a ground electrode was inserted into the opposite lower leg. A total of 20 consecutive stimulation trials were performed and the incidence of MEP response was recorded for each individual trial. In parallel, response averaging was used to calculate the response latency and amplitude for each animal across all 20 trials. Finally, compound muscle action potentials (CMAPs) were recorded to test for changes in peripheral conduction. We used a custom-made bipolar stimulating electrode placed percutaneously to the sciatic nerve at the sciatic notch (proximal CMAPs) or at the tarsal tunnel (distal CMAPs). Proximal CMAPs were recorded via subcutaneous electrode at the gastrocnemius and reference at the Achilles tendon. Distal CMAPs were measured at the footpad with reference at the fibular side of the foot. Supramaximal stimulation was used to generate a stable CMAP. Recordings were filtered through a 2 Hz to 20 kHz bandpass filter and onset latency, amplitude, area under the negative peak, and duration of the negative peak were measured.

*In vivo immunodepletion of CD8^+^ T cells.*

Wildtype B10Q mice were immunodepleted of CD8^+^ T cells via weekly intraperitoneal injections of 0.5 mg of the anti-CD8 2.43 clone (ATCC, No. TIB 210) from 45 dpi to 90 dpi. Control animals were treated in parallel with 0.5 mg of the control antibody OKM1 (ATCC, No. CRL 8026). Antibodies were prepared by ammonium sulfate precipitation from 2.43 and OKM1 hybridoma supernatants.

*Statistics.*

All data are presented as mean ± 95% confidence interval (CI). Statistical tests for every analysis are described in detail in Supplementary Table 1. Normality was used when appropriate to determine whether parametric or non-parametric tests were employed. All calculations were performed using SigmaPlot 11 (Systat Software, Chicago, IL). Every experiment was repeated a minimum of 3 times on separate cohorts.

*References.*

1. Rodriguez M (1991) Immunoglobulins stimulate central nervous system remyelination: electron microscopic and morphometric analysis of proliferating cells. Lab Invest 64: 358-370.

2. Howe CL, Adelson JD, Rodriguez M (2007) Absence of perforin expression confers axonal protection despite demyelination. Neurobiol Dis 25: 354-359.

3. Deb C, Howe CL (2008) NKG2D contributes to efficient clearance of picornavirus from the acutely infected murine brain. J Neurovirol 14: 261-266.

4. Deb C, Lafrance-Corey RG, Zoecklein L, Papke L, Rodriguez M, et al. (2009) Demyelinated axons and motor function are protected by genetic deletion of perforin in a mouse model of multiple sclerosis. J Neuropathol Exp Neurol 68: 1037-1048.

5. Sango K, McDonald MP, Crawley JN, Mack ML, Tifft CJ, et al. (1996) Mice lacking both subunits of lysosomal beta-hexosaminidase display gangliosidosis and mucopolysaccharidosis. Nat Genet 14: 348-352.
